# Effects of Broad-Spectrum Antibiotic (Florfenicol) on Resistance Genes and Bacterial Community Structure of Water and Sediments in an Aquatic Microcosm Model

**DOI:** 10.3390/antibiotics11101299

**Published:** 2022-09-23

**Authors:** Tengyue Zhang, Yuexia Ding, Jinju Peng, Yue Dai, Shuaishuai Luo, Wenchao Liu, Yi Ma

**Affiliations:** 1 Department of Veterinary Medicine, College of Coastal Agricultural Sciences, Guangdong Ocean University, Zhanjiang 524088, China; 2 Department of Animal Science, College of Coastal Agricultural Sciences, Guangdong Ocean University, Zhanjiang 524088, China; 3 Maoming Branch, Guangdong Laboratory for Lingnan Modern Agriculture, Maoming 525000, China

**Keywords:** florfenicol, antibiotic resistance genes, bacterial community structure, 16S rDNA, aquatic microcosm

## Abstract

This study evaluates the effects of a broad-spectrum antibiotic (florfenicol) on antibiotic resistance genes (ARGs) and bacterial community structure in aquatic environments. We constructed an indoor aquatic microcosm model, adding different concentrations of florfenicol (0.1, 1, 10, 100 mg L^−1^), and water and sediment samples were collected after 0, 7, 30, and 60 days. qPCR and 16S rDNA amplicon sequencing were used to study the changes in the ARGs and bacterial community structure of the collected samples. The results show that the inclusion of florfenicol resulted in an increased abundance of the *floR* and *optrA* genes. Adding 100 mg L^−1^ florfenicol to the water increased the abundance of *optrA* gene copies with the maximum on the Day 7, and increased the abundance of *floR* gene copies with the maximum on Day 30. Adding 100 mg L^−1^ florfenicol to the sediment increased the abundance of *floR* and *optrA* genes by one order of magnitude on Day 60. Meanwhile, the average number of operational taxonomic units (OTUs) in the water samples was 257, and the average number of OTUs in sediment samples was 823. The bacterial community diversity and richness in sediments were higher than those in water. The difference between the maximal and minimal values of the Shannon diversity index in the water and sediment samples was 4.36 and 1.95, respectively. The effect of florfenicol on the bacterial community structure in water was much higher than that in sediment. At 30 days, the diversity index and richness index of the florfenicol treatment groups with 1 and 10 mg L^−1^ concentrations began to increase; at 60 days, the diversity and richness indices of the 100 mg L^−1^ florfenicol treatment group began to increase. The samples at the same sampling time in the sediments clustered closer together. The results of this study provide a scientific basis for guiding the rational use of florfenicol in aquaculture, maintaining a healthy and stable microecological environment in aquaculture, and provide theoretical data for environmental ecological risk assessment and safety management caused by microbial resistance under the abuse of florfenicol.

## 1. Introduction

Antibiotics have played a vital role in human medical care and animal production in the past few decades. China is a large country in animal husbandry, and most of the produced antibiotics are used on animals [1,2,3]. According to the FAO report in 2020, the scale of global aquaculture is increasing, and China’s aquaculture industry is also at a stage of rapid development [4,5]. The use of antibiotics in intensive farming is currently universal. Florfenicol is one of the antibiotics approved by FAO for use in aquaculture and one of the most widely used antibiotics in the aquacultural industry [6]. Florfenicol binds to Site A on the 50S large subunit of the bacterial ribosome, thereby affecting the transpeptidase reaction of peptidyl transferase, inhibiting the production of bacterial proteins [7]. However, florfenicol has stable physical and chemical properties, it is not easy to degrade, and a large amount of unabsorbed florfenicol is continuously discharged, which leads to its persistent existence in the environment [8]. Different concentrations of florfenicol residues have been detected in coastal waters, sediments, and organisms [9]. After 6 months of antibiotic treatment in all salmon farms in Puyu Huapi Fjord, Chile, the detected concentration of florfenicol was as high as 23.1 ng·L^−1^ in aquacultural water [10]. The detected concentration of florfenicol in the Yangtze River basin was 89.5 ng·L^−1^, and the highest detected concentrations in the Three Gorges Reservoir and Songhua River basin were 46.6 and 3.3 ng·L^−1^, respectively [11,12]. Furthermore, the residual concentration of florfenicol was as high as 11 mg·L^−1^ in the waters around the Dalian Bay aquacultural farms in China [13]. Chen and Zhou [14] found that the highest concentrations of florfenicol in the water and sediment of the Huangpu River were 241.1 and 1.3 μg·kg^−1^, respectively. The drug residues caused by the irregular use of antibiotics can induce the emergence of antibiotic resistance genes (ARGs) [15]. ARGs are released into the environment after the death of microorganisms and are protected from deoxyribonuclease (DNAse) by tightly binding onto soil particles, and their persistent residual properties render them far more harmful than antibiotics themselves [16]. The *floR* gene is the earliest discovered and currently the most widespread florfenicol resistance gene, and the *optrA* gene can mediate oxazolidinones and amide alcohol drugs; once these genes spread into the human body through the food chain, they pose a huge threat to human public health [17].

Microorganisms are an important part of the ecosystem, and participate in the material cycle and energy flow in nature [18]. Florfenicol is a broad-spectrum antibiotic that can inhibit a variety of bacteria; therefore, the remaining antibiotics in the environment inevitably impact microorganisms [19]. When the antibiotic concentration in a water environment exceeds the limit, the activity of microorganisms is inhibited, and the community structure of microbiota changes accordingly, thus destroying the stability and resilience of the ecosystem [20]. Florfenicol is globally the most widely used antibiotic for aquaculture, and the amount of florfenicol used in China is as high as 10,000 tons per year [21]. Relevant studies show that florfenicol treatment has shifted the structure of the microbiota and reduced its biodiversity by acting as a strong stressor. Florfenicol treatment promotes the proliferation of florfenicol-resistant genes and induces mutation-driven resistance [22]. However, there are fewer reports on the effects of florfenicol on ARGs, and the microbial community structure of water and sediments in an aquatic microcosm model.

In this study, an indoor aquatic microcosm model was constructed to simulate an aquatic ecosystem, and florfenicol antibiotics were added exogenously to explore changes in the abundance of ARGs after different concentrations of florfenicol had entered the water environment, and their impact on the bacterial community structure, diversity, and abundance. The present study can provide a theoretical basis for the scientific use of florfenicol in aquaculture to maintain a healthy ecological environment.

## 2. Results

### 2.1. Abundance of Florfenicol ARGs

**Figure 1:**
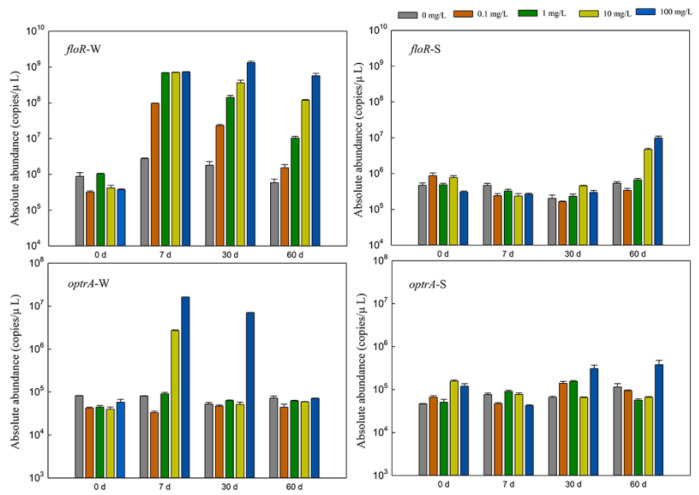
Effects of different concentrations of florfenicol on the abundance of antibiotic resistance genes (ARGs) in an aquatic microcosm model. W, water; S, sediments.

As shown in Figure 1, the abundance of the *floR* gene in the water body ranged from 3.24 × 10^5^ to 1.33 × 10^9^ copies·μL^−1^. On Day 7, after the addition of florfenicol, the abundance of the *floR* gene was one order of magnitude higher than that of the control group, and 0.1 mg·L^−1^ of florfenicol could significantly increase the abundance of the *floR* gene. The abundance of the *floR* gene decreased with time. The abundance of the *floR* gene in sediments ranged from 1.63 × 10^5^ to 9.86 × 10^6^ copies·μL^−1^. The abundance of the *floR* gene was one order of magnitude higher on Day 60 after the addition of 10 and 100 mg·L^−1^ florfenicol. The abundance of the *optrA* gene in water ranged from 3.34 × 10^4^ to 1.62 × 10^7^ copies·μL^−1^. On Day 7, after 10 and 100 mg·L^−1^ florfenicol supplementation, the abundance of the *optrA* gene increased by 1–2 orders of magnitude. With the change in time, the abundance of the *optrA* gene showed a downward trend, and the abundance of the *optrA* gene in the samples added with florfenicol at 60 days was basically the same as that in the control group. The abundance of the *optrA* gene in the sediments ranged from 4.24 × 10^4^ to 3.81 × 10^5^ copies·μL^−1^, and the abundance of the *optrA* gene increased by one order of magnitude on Days 30 and 60 after the addition of 100 mg·L^−1^ fluorobenzene. According to the statistical analysis results, concentrations of florfenicol and time significantly affected the abundance of ARGs in an aquatic microcosm model (*p* < 0.001).

### 2.2. Analysis of OTUs

**Figure 2:**
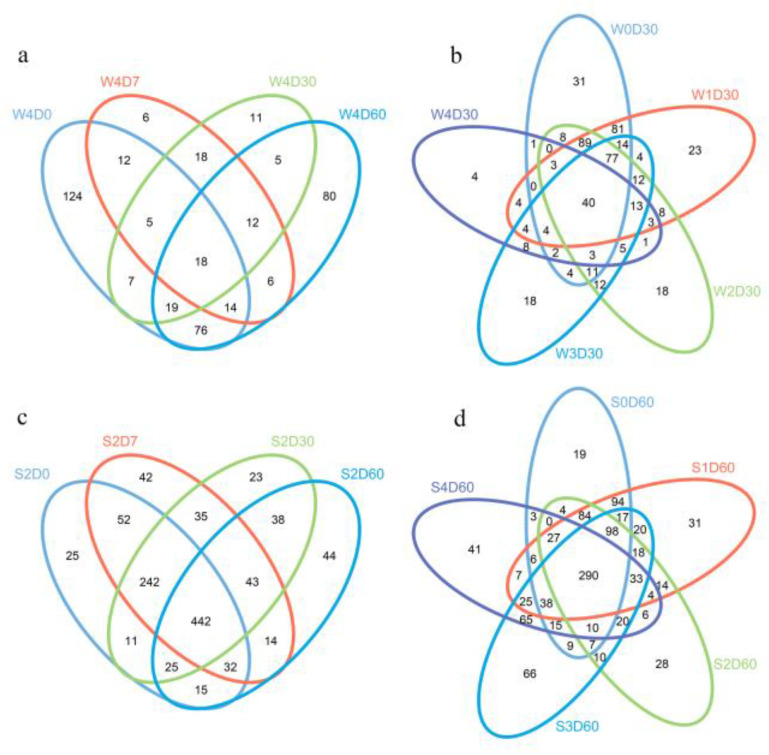
Effects of different concentrations of florfenicol on operational taxonomic units (OTUs) of bacteria (Venn diagram) in an aquatic microcosm model. (**a**) Effect of sampling time on OUTs of bacteria in group W4 samples. (**b**) Effects of different concentrations of florfenicol on OTUs of bacteria in water samples at 30 days. (**c**) Effect of sampling time on the OUTs of bacteria in group S2 samples. (**d**) Effects of different concentrations of florfenicol on OTUs of bacteria in sediment samples at 60 days. W, water; S, sediments. Differently colored circles represent different samples, and the numbers represent the number of unique OTUs in each sample or the number of common OTUs to all samples.

After the quality optimization of the original data, the number of valid sequences obtained in water samples was 31,046,103,252, and the number of valid sequences in the sediment samples was 31,570–74,821. After OTU cluster analysis, a total of 580 OTUs were obtained from the water samples, with an average of 257 OTUs per sample, of which the W4D7 group had the fewest OTUs, 91; a total of 1235 OTUs were obtained from the sediments, and the average number of OTUs per sample was 823, of which the S4D60 group had the fewest OTUs, 590. According to the out cluster analysis results, a Venn diagram was drawn, and there were 7 OTUs in water samples and 224 OTUs in sediment samples. As shown in Figure 2, the number of common OTUs was the smallest when the concentration of florfenicol in the water samples was 100 mg·L^−1^ (Figure 2a), and the number of common OTUs in the sediment samples was the smallest when the concentration of florfenicol was added at 1 mg·L^−1^ (Figure 2b). The 30-day water samples shared the fewest OTUs (Figure 2c), and the 60-day sediment samples shared the fewest OTUs (Figure 2d).

**Figure 3:**
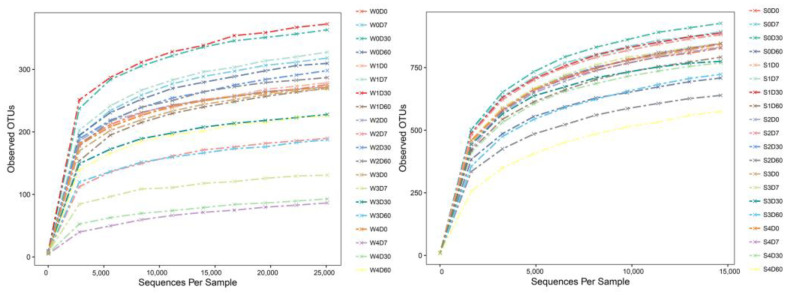
Effects of different concentrations of florfenicol on dilution curve of operational taxonomic units (OTUs) of bacteria in an aquatic microcosm model. W, water; S, sediments.

Sequences were randomly sampled, and a dilution curve was constructed with the number of valid sequences drawn from the sample (sequences per sample) as the abscissa and the number of (observed) OTUs as the ordinate. As presented in Figure 3, as the number of extracted sequences increased, the number of detected OTUs increased and gradually became flat, indicating that the sequencing tended to be saturated, the sampling was basically reasonable, and that the sequencing results contained the most microbial groups. This can more realistically reflect the microbial community structure in the sample through the addition of 100 mg·L^−1^ fluorobenzene.

### 2.3. Alpha Diversity Index Analysis

The community richness index mainly includes the Chao1 and ACE indices, and the bacterial community diversity index mainly includes the Shannon and Simpson indices. As shown in Table 1, with the increase in drug concentration, the species richness and diversity of the water samples showed a downward trend; with the change in time, the community richness and diversity were first suppressed and then gradually recovered. The diversity index of the W2 and W3 groups increased on Day 30, while the diversity index of the W4 group began to increase on Day 60, and the minimal value of the diversity index appeared in the W4 group. As shown in Table 2, among the sediment samples, only the S4 and D60 groups exhibited the inhibitory effect of florfenicol on the bacterial species richness and diversity, and the smallest value of the diversity index appeared in the 60-day sediment samples when the concentration of florfenicol was added at 100 mg·L^−1^.

### 2.4. Bacterial Community Structure Analysis

**Figure 4:**
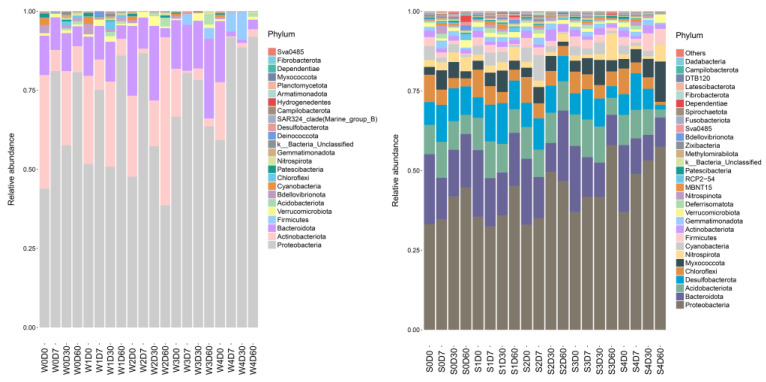
Effects of different concentrations of florfenicol on phylum-level bacterial abundance in an aquatic microcosm model. W, water; S, sediments.

Species relative abundance analysis was performed at the phylum level, and a histogram of species information is indicated in Figure 4. A total of 24 gates were obtained for the water bodies, and 40 gates for sediments. The dominant bacteria in the water were *Proteobacteria*, *Actinobacteriota* and *Bacteroidota*, and the dominant bacteria in the sediment were *Proteobacteria*, *Bacteroidota, Desulfobacteria* (*Desulfobacterota*), and *Acidobacteriota*, *Myxococcota*. *Proteobacteria* were the dominant bacteria in all samples, with relative abundances ranging from 39% to 92% in water, and 33% to 57% in sediments. Compared with the control group, the relative abundance of *Proteobacteria* increased significantly, while the relative abundance of *Actinobacteria* and *Bacteroidetes* decreased significantly in the water samples. Significant changes in the microbial community structure of the sediments appeared on Days 30 and 60. The relative abundance of *Proteobacteria* and *Myxococcota* increased, and the relative abundance of *Bacteroidota, Desulfobacterota, Acidobacteriota,* and *Chloroflexi* decreased. The relative abundance of *Firmicutes* in the water samples of the control group was 0, while the relative abundance in the W4D7 and W4D30 samples was 6.1% and 8.9%, respectively.

**Figure 5:**
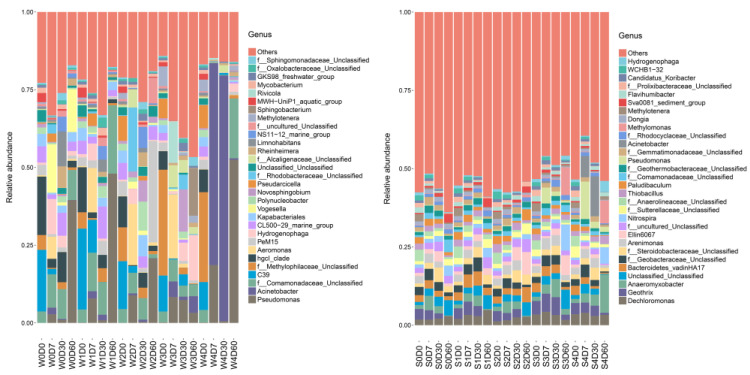
Effects of different concentrations of florfenicol on genus-level bacterial abundance in an aquatic microcosm model. W, water; S, sediments.

At the genus level, a total of 257 genera were obtained from the water samples, with the number of each group ranging from 47 to 181; a total of 435 genera were obtained from the sediments, with the number of each group ranging from 270 to 367. The top 30 genus-level species were selected to draw a histogram. As shown in Figure 5, the main dominant bacterial genera in the water without florfenicol were *hgcI-clade*, *CL500-29-marine-group*, *PeM15*, *Bacteroidota*, and *Kapabacteriales*. On Day 7, florfenicol had a significant effect on the bacterial community structure in the water. With the increase in florfenicol concentration, the relative abundances of *Vogesella*, *Hydrogenophaga*, *Comamonadaceae*, and *Cellvibrio* in *Proteobacteria* were decreased. The dominant bacterial genera in the sediments were *Dechloromonas*, *Geothrix*, *Anaeromyxobacter*, *Bacteroidetes-vadinHA17*, *Geobacteraceae, Steroidobacteraceae*, *Arenimonas*, and *Ellin6067*. Compared with water, the bacterial diversity in the sediments was higher, the community structure was more complex and stable, and it was stronger in resisting antibiotic stress.

### 2.5. Multisample Comparative Analysis

**Figure 6:**
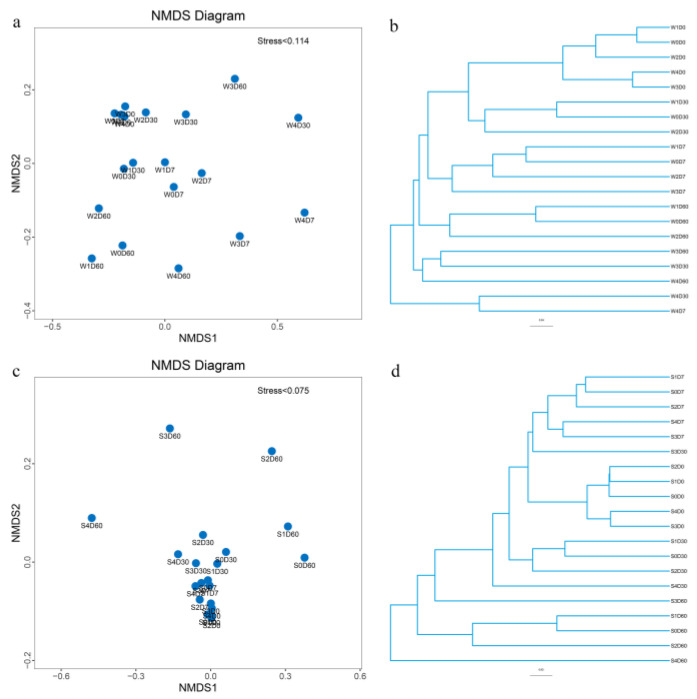
Nonmetric multidimensional scaling method (NMDS) analysis reveals the degree of similarity and difference of bacteria between different samples in an aquatic microcosm model. W, water; S, sediments. (**a**) The results of the NMDS analysis of water samples; (**b**) The results of the UPGMA analysis of water samples; (**c**) The results of the NMDS analysis of sediment samples. (**d**) The results of the UPGMA analysis of sediment samples.

The nonmetric multidimensional scaling method (NMDS analysis) and unweighted pair group method with arithmetic mean (UPGMA) clustering were used to express the degree of similarity and difference between different samples. The results are shown in Figure 6. When stress is less than 0.2, NMDS analysis can accurately reflect the degree of difference between samples; water samples with similar microbial community structure were clustered together in cluster analysis. W4D7 and W4D30W, and W3D30 and W3D60 were clustered together, and NMDS analysis was farther away from other treatment groups. S2D60, S3D60, and S4D60 in the sediment samples were significantly separated from other treatment groups, cluster analysis shows obvious time differences, and the treatment groups with different sampling times had farther clustering distances regarding antibiotic stress.

### 2.6. Correlation Analysis between Bacterial Communities and ARGs

**Figure 7:**
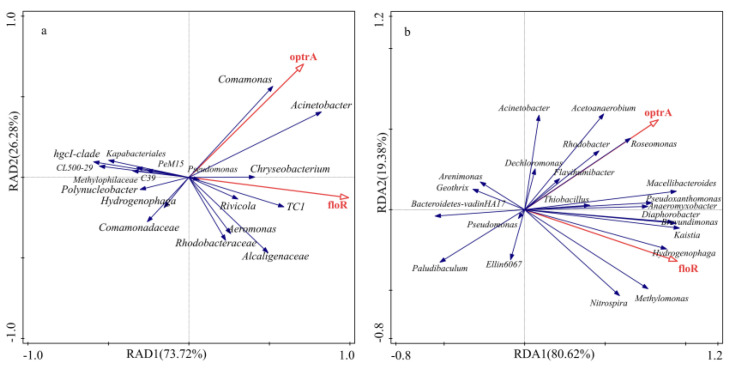
Correlation analysis between bacterial communities and ARGs in an aquatic microcosm model. W, water; S, sediments; (**a**) The results of the redundancy analysis of water samples; (**b**) The results of the redundancy analysis of sediment samples.

Taking ARGs as environmental factors, combined with the microbial community structure of each sample, bacteria with higher relative abundance were selected as samples, and redundancy analysis (RAD) was used to study the correlation between bacterial community structure and ARGs. The results of redundancy analysis of water samples are shown in Figure 7a. Spindles 1 and 2 explain 73.72% and 26.28% of the bacterial community structure and ARGs parameters, respectively. Among them, genera *Comamonas*, *Acinetobacter*, *Chryseobacterium*, *Pesudomonas*, and other genera in the bacterial community were positively correlated with the *optrA* resistance gene, and *TC1*, *Rivicola*, *Acinetobacter, Chryseobacterium*, *Pseudomonas*, *Alcaligenaceae*, and other genera were positively correlated with the *floR* resistance gene. The results of the redundancy analysis of sediment samples are shown in Figure 7b. Spindles 1 and 2 explain 80.62% and 19.38% of the bacterial community structure and ARGs parameters, respectively. In the bacterial community, genera *Roseomonas*, *Rhodobacter*, *Flavihumibacter*, *Acetoanaerobium*, *Macellibacteroides* were positively correlated with the *optrA* resistance gene, and genera such as *Hydrogenophaga, Kaistia, Brevundimonas*, *Methylomonas*, *Nitrospira*, and *Diaphorobacter* were positively correlated with the *floR* resistance gene.

## 3. Discussion

Under antibiotic pressure selection, bacteria in aquacultural environments develop drug resistance and ARGs, thereby destroying the environmental microecological balance [23]. Aquatic environments are vulnerable to contamination through the use of antibiotics in aquacultural wastewater, and the increased selective pressure caused by the extensive use of florfenicol in aquaculture has accelerated the development and spread of bacterial resistance to this antibiotic, resulting in potential public health risks [24,25]. Among the reported resistance genes of florfenicol, the *floR* gene mainly mediates the resistance of Gram-negative bacteria to florfenicol [26]. The *optrA* gene is involved in bacterial resistance to amide alcohols and oxazolidinones; oxazolidinones (e.g., linezolid and tedizolid) are currently only approved for human infection by superbugs such as methicillin-resistant *Staphylococcus aureus* and vancomycin-resistant *Enterococcus*, so they are the “last line of defense” against pathogenic bacteria [27]. In this experiment, the changes in florfenicol resistance genes *floR* and *optrA* were determined by qPCR. The results show that the addition of florfenicol had a greater impact on the abundance of ARGs in water, and 0.1 mg·L^−1^ florfenicol could increase *floR* gene abundance by 1–3 orders of magnitude, while 10 and 100 mg·L^−1^ florfenicol increased *optrA* gene abundance by 1–2 orders of magnitude. With the deposition at higher florfenicol concentration and longer time, the abundance of ARGs began to increase. In addition, the abundance of both ARGs in the water body decreased with time, and the abundance of the *optrA* drug resistance gene recovered to the control level at 60 days. A previous study showed that the *floR* gene is found on both chromosomes and plasmids, and plasmids carrying the *floR* gene can be spread between bacteria [28]. Miranda et al. [26] also isolated a large number of florfenib-resistant strains from two commercial scallop hatcheries in Chile carrying the *floR* gene. Furthermore, Fan et al. [29], and Torres et al. [30] found that the *optrA* resistance gene exists in both *Staphylococcus* and *Enterococcus*. Wang et al. [31] observed that the addition of florfenicol to soil significantly affected the four resistant genotypes of aminoglycosides, β-lactamases, tetracyclines, and trimethoprim, while the resistance to the amide alcohol genotype had no significant effect. However, other relevant findings suggested that the abundance of resistance genes did not decrease after antibiotics had been discontinued in aquaculture, but remained at a high level [32]. The inconsistencies with the present study may have been caused by the environmental differences and different host species of ARGs. They may also be related to the amount and period of used antibiotics [33].

Water environmental safety is closely associated with the bacterial community structure; when the water environment changes, the bacterial community structure is also altered [34]. The effects of antibiotics on microbial communities are mainly community diversity, abundance, composition, and function [35]. According to a previous report, the impact of antibiotics on the bacterial community structure can be roughly divided into three stages: the first stage has no obvious effect on bacterial abundance, but affects its physiological activity; the second stage has a significant inhibitory effect on bacterial abundance, and the bacterial community difference increases; in the third stage, the bacterial community structure basically returns to the control level [36]. The in vitro minimal inhibitory concentration (MIC) of florfenicol against common pathogens is 0.3–1.6 μg·mL^−1^. In the present study, 0.1 mg·L^−1^ florfenicol in the water environment did not affect the diversity and abundance of bacterial communities, but promoted the growth of some specific bacteria; 100 mg·L^−1^ florfenicol had a dramatic effect on the bacterial community structure, significantly reducing the diversity and abundance of the bacterial communities. The effect of florfenicol on bacterial diversity gradually decreased with time. When the addition of florfenicol was 100 mg·L^−1^, the bacterial community structure in the sediment was disturbed, and the community diversity and richness decreased. The relative abundance of Acinetobacter strains dominated after supplementing 100 mg·L^−1^ florfenicol, including *Acinetobacter variabilis*, *Acinetobacter soli,* and *Acinetobacter baylyi*. All three *Acinetobacter* species are resistant to carbapenems [37,38,39]. The study of Yong-Ji et al. [40] showed that florfenicol significantly affects the environmental bacterial community structure, leading to a complete subversion of the microbial community. Liu et al. [41] suggested that florfenicol is mainly degraded and eliminated in the form of a prototype in an aquacultural environment, most of the drugs persist in the water until degraded, and the content of the drugs in the sediment is less. This is consistent with the changing trend of the results in our experiment. In addition, some studies indicated that sediments have a strong adsorption capacity for certain antibiotics. With changes in the external environment, antibiotics adsorbed in sediments may be resolved and released into waters [42], which can also explain our findings.

ARGs affect the diversity index of soil bacterial communities [43]. Meanwhile, microbes serve as hosts for ARGs, and changes in their community structure are related to the expression of ARGs [44]. The correlation analysis of this study shows that *Acinetobacter*, *Chryseobacterium*, and *Pseudomonas* were positively correlated with the *optrA* and *floR* genes in water; these three bacteria are common opportunistic pathogens and may also be the potential host bacteria of florfenicol resistance genes. *Acinetobacter* has the ability to acquire OXA-type carbapenemases and metallo-β-lactamases, and develop rapid resistance to new antibiotics; therefore, it can become the dominant genus under antibiotic stress [45]. *Chryseobacterium* is widespread in water, soil, and hospital environments. *Chryseobacterium* is resistant to a variety of antibiotics, such as lactams, aminoglycosides, and quinolones [46,47,48]. Fu et al. [49] isolated multidrug-resistant *Chryseobacterium* sp. POL2 from livestock and poultry farming wastewater. The integrated engagement element of this strain can confer new drug resistance and pathogenicity to the host bacteria, allowing for the host bacteria to rapidly adapt to changing ecological niches, increasing the risk of transmission and infection. *Pseudomonas* has a variety of environmental adaptation mechanisms and is widely present in the environment [50]. In both veterinary and human medicine, resistance to commonly used antipseudomonal antibiotics such as carbapenems, cephalosporins, fluoroquinolones, and aminoglycosides was reported [51,52,53]. *Pseudomonas* has multiple drug-resistance mechanisms, including target gene mutations, changes in the expression of genes encoded by the efflux pump system, the production of inactivating enzymes, and the formation of biofilms [54,55]. The selection pressure of antibiotics makes these potential pathogenic bacteria acquire resistance genes and become dominant genera, accelerating the spread of ARGs and the risk of disease outbreaks. Hence, avoiding the misuse of broad-spectrum antibiotics such as florfenicol in aquaculture is imperative.

## 4. Materials and Methods

### 4.1. Reagents

Lake surface water (1–10 cm) and lake surface sediments (1–10 cm) in Guangdong Ocean University (Zhanjiang, China) were collected, and the debris was removed to construct an aquatic microcosm. Florfenicol (13021322) was purchased from North China Pharmaceutical Co., Ltd. (Hebei, China). Water DNA Kit (D5525), Soil DNA Kit (D5625), and MicroElute Gel Extraction Kit (D6294) were purchased from Omega Bio-Tek Company (Guangzhou, China). TIANprep Midi Plasmid Kit (DP106-02) was purchased from Tiangen Biochemical TechnologyCo., Ltd. (Beijing, China). pMD^TM^ 19-T Vector Cloning Kit (6013) was purchased from Bao Bioengineering Co., Ltd. (Dalian, China). ChamQ Universal SYBR qPCR Master Mix (Q711- 02) was purchased from Nanjing Novizan Biotechnology Co., Ltd. (Nanjing, China). DH5α competent cells (B528413), Real-Time PCR Plates, 96-Well Transparent, Non Skirted (F603101) and primers were purchased from Sangon Bioengineering Co., Ltd. (Shanghai, China).

### 4.2. Experimental Design and Sampling

The indoor aquatic microcosm model was constructed to simulate the aquatic ecosystem. The collected lake water and sediment were picked out and packed in a transparent 50 × 40 × 30 cm plastic box. A total of 15 aquatic microcosm models were constructed. The sediment thickness of the group was about 10 cm, and the water body was about 30 L. After the aquatic microcosm system had basically stabilized, a florfenicol solution was added to the water body to concentrations of 0, 0.1, 1, 10 and 100 mg·L^−1^ (the numbers are water samples W0–W4 and sediment samples S0–S4), placed at room temperature of 25 ± 3 °C, and the water samples were collected at four points at 0, 7, 30, and 60 days (D0, D7, D30, D60) at a distance of about 10 cm from the water surface (W group) and surface sediment (1–5 cm) samples (group S); the sediment samples were mixed and centrifuged to remove the supernatant. The experiment was divided into 5 groups, each with 3 replications.

### 4.3. Detection of ARGs

The SYBR-primer method was used to determine the abundance of ARGs on the CFX Connect Real-Time System instrument (BIO-RAD). Primer-related information is shown in Table 3 and was based on the previous studies [56,57]. The purified product after PCR amplification was ligated into a 19-T vector, and the ligated product was transformed into competent *E. coli*. Positive clones were selected, plasmids were extracted from them, and the plasmid concentration was determined using a NanoDrop microspectrophotometer. The known plasmid concentration was diluted with fivefold gradient concentration, the logarithmic value of copy number was used as the abscissa, and the C_T_ value was used as the ordinate to draw a standard curve. Three repetitions were performed for each group, and the average was taken.

DNA copy number (copies·μL^−1^) = [C × 10^−9^ × 6.02 × 10^23^]/[L × 660]

where C is the plasmid concentration (ng·μL^−1^), and L is the number of bases of the cloned product (bp). The qPCR reaction system was: 10 µL SYBR, 1 µL DNA template, 1 µL upstream and downstream primers (10 mol·L^−1^) each, and 8 µL ddH_2_O. The qPCR reaction program was: 94 °C, 5 min; 94 °C for 30 s, annealing for 30 s (annealing temperature is shown in the table), 72 °C for 30 s, 39 cycles; fluorescence was collected after each cycle. From 60 to 95 °C, the fluorescence was collected every 0.5 °C to generate the melting curve to test the specificity of the amplification results.

### 4.4. 16S rDNA Sequencing

The extraction of the total microbial genome DNA from water and sediments was according to the instructions of the commercial kits. The concentration and purity of the extracted DNA were determined with a nanodrop UV–vis spectrophotometer. The DNA samples extracted from each group of the 3 replicates were mixed into one sample and sent to Suzhou Jinweizhi Biotechnology Co., Ltd. (Suzhou, China) for the Illumina MiSeq paired-end sequencing of the V3–V4 amplicon regions of 16S rDNA. The sequencing details were based on a former report [58].

### 4.5. Statistical Analysis

QIIME 1.9.1 was used to optimize the high-throughput sequencing raw data: splicing the overlapping regions at the ends of the sequences, removing sequences with a length of less than 200 bp, and removing the chimeric sequences to obtain valid data. Operational taxonomic unit (OTU) cluster analysis was performed according to 97% similarity. Species taxonomic annotation was performed using the Silva 138 16S rDNA database. Excel 2016 was used to analyze the abundance of ARGs, QIIME 1.9.1 software was used for α diversity index analysis, R language was used for β diversity analysis, and CANOCO 5.0 software was used to analyze the correlation between ARGs and the community structure. GLMs followed by multiple comparisons using Duncan’s test were applied to test the effects of concentrations of florfenicol, time, and their interactions on the abundance of ARGs. Statistical analyses were performed with SAS 9.2 (SAS Institute Inc., Cary, NC, USA).

## 5. Conclusions

In this study, the addition of florfenicol increased the abundance of the *floR* and *optrA* genes by orders of magnitude. The bacterial community diversity and richness in sediment were higher than those in water. The addition of florfenicol reduced the diversity and richness of the bacterial community, and the effect on the bacterial community structure in water was much higher than that in the sediment. The richness and diversity of the bacterial communities gradually recovered over time. High concentrations of florfenicol break the original community structure and significantly increase the relative abundance of drug-resistant bacteria. Therefore, to reduce the risks of environmental safety and public health that are posed by florfenicol, the standardized use of broad-spectrum antibiotics such as florfenicol in aquaculture, and closely monitoring and unraveling alternatives to antibiotics are necessary. 

## Figures and Tables

**Table 1:** Alpha diversity index of bacteria in water.

Sample	OTUs	ACE	Chao1	Shannon	Simpson
W0D0	279	320.58	328.79	5.99	0.97
W1D0	283	328.62	334.04	5.86	0.96
W2D0	275	293.72	294.33	5.96	0.97
W3D0	273	300.98	302.64	5.05	0.90
W4D0	275	296.88	304.53	5.36	0.92
W0D7	323	350.91	349.28	5.48	0.95
W1D7	333	376.69	396.58	5.85	0.96
W2D7	194	219.66	217.62	4.90	0.94
W3D7	135	164.48	164.25	4.62	0.93
W4D7	91	152.33	137.75	2.59	0.73
W0D30	368	396.82	397.13	6.50	0.98
W1D30	379	424.92	438.11	6.95	0.99
W2D30	303	349.49	340.50	6.12	0.97
W3D30	231	261.72	260.75	5.73	0.96
W4D30	95	127.48	131.11	2.96	0.76
W0D60	314	351.89	351.60	5.31	0.91
W1D60	274	308.32	308.14	4.05	0.85
W2D60	291	308.85	307.53	4.93	0.89
W3D60	191	230.08	217.25	5.24	0.95
W4D60	230	261.66	267.05	4.13	0.84

**Table 2:** Alpha diversity index of bacteria in sediments.

Sample	OTUs	ACE	Chao1	Shannon	Simpson
S0D0	841	922.03	955.24	8.37	0.99
S1D0	847	918.58	939.26	8.41	1.00
S2D0	844	928.59	941.04	8.36	0.99
S3D0	857	953.93	977.28	8.37	0.99
S4D0	858	944.58	952.10	8.39	0.99
S0D7	904	982.03	996.36	8.67	1.00
S1D7	908	1002.84	1017.90	8.61	1.00
S2D7	902	1004.74	1049.35	8.58	1.00
S3D7	862	941.05	954.55	8.28	0.99
S4D7	860	944.85	947.53	7.83	0.98
S0D30	940	1016.88	1027.78	8.67	1.00
S1D30	900	970.75	967.22	8.58	1.00
S2D30	859	942.94	965.21	8.31	0.99
S3D30	785	825.71	829.69	8.31	0.99
S4D30	782	848.19	860.47	7.87	0.99
S0D60	721	810.66	817.40	7.95	0.99
S1D60	806	896.28	908.75	8.16	0.99
S2D60	653	748.21	733.68	7.53	0.99
S3D60	741	865.00	877.07	7.57	0.99
S4D60	590	727.29	731.81	6.72	0.98

**Table 3:** Primer information of antibiotic resistance genes (ARGs) for qPCR.

Genes	Sequences (5′-3′)	Product size (bp)	Annealing Temperature (°C)
*floR*	F: GCGATATTCATTACTTTGGC	425	54
	R: TAGGATGAAGGTGAGGAATG		
*optrA*	F: CTTATGGATGGTGTGGCAGC	310	59
	R: CCATGTGGTTTGTCGGTTCA		

## Data Availability

16S rDNA sequencing data were uploaded to the repository (National Center for Biotechnology Information, NCBI) that can be found at http://www.ncbi.nlm.nih.gov/bioproject/841751 (accession no., PRJNA841751).
